# Perceptions of health professionals on structure and process of stroke rehabilitation in Ghana

**DOI:** 10.4102/ajod.v12i0.1116

**Published:** 2023-04-03

**Authors:** Tawagidu Mohammed, Gifty G. Nyante, Joyce D. Mothabeng

**Affiliations:** 1 Department of Physiotherapy, School of Healthcare Sciences, University of Pretoria, Pretoria, South Africa; 2 Department of Physiotherapy, School of Biomedical and Allied Health Sciences, University of Ghana, Accra, Ghana

**Keywords:** stroke, stroke rehabilitation, structure, process, healthcare professionals, Ghana

## Abstract

**Background:**

Ensuring quality in the structure and process of stroke rehabilitation helps to attain a good outcome. However, knowledge on this is limited in resource-constrained settings such as Ghana.

**Objectives:**

This study aimed to explore healthcare professionals’ (HCPs) views and experiences of the structure and process of stroke rehabilitation in three selected hospitals in Ghana.

**Method:**

A qualitative study was carried out involving 26 HCPs directly involved in stroke rehabilitation from three selected hospitals in the Greater Accra Region of Ghana representing the different levels of healthcare. Interviews were conducted using an interview guide to understand participants’ views and experiences of the structure and process of stroke rehabilitation. Interview transcripts were analysed using thematic analysis.

**Results:**

HCPs reported limitations with the structure of stroke rehabilitation with regards to the availability of rehabilitation units, bed capacity, approach to care, availability of protocol, staff capacity development and payment systems. With respect to the process of rehabilitation, the primary and secondary level hospitals were found not to have computed tomography (CT) and magnetic resonance imaging (MRI) scanning equipment. Participants also reported limitations with discharge planning, basis for discharge and post-discharge care across all three hospitals.

**Conclusion:**

This study found limitations in the current structure and process of stroke rehabilitation, which when given some considerations for improvement, can help improve the quality of care and thereby improve the outcome of stroke patients in Ghana.

**Contribution:**

This study provided data which helps to assess the quality of stroke rehabilitation in Ghana.

## Introduction

Stroke continues to be of major public health concern globally, as stroke is the second leading cause of death and the third leading cause of disability (Katan & Luft 2018). About 70% of strokes and 87% of both stroke-related deaths and disability-adjusted life years occur in low- to middle-income countries (LMICs) such as sub-Saharan African countries (Feigin et al. 2021). Ghana, which is also categorised under LMICs is located in west Africa and has a population of about 25 million (Drislane et al. 2014). Ghana is rapidly undergoing epidemiological transitions of diseases and the burden of disease has now shifted from communicable to non-communicable diseases over the last few years (Sanuade et al. 2019). One cardiovascular disease that is on the rise is hypertension and this is a major risk factor for stroke. In Ghana, the mortality rate of stroke is about 40% (Baatiema et al. 2017a). Stroke has now become the major cause of adult medical admissions in Ghana and a major cause of adult disability (Agyei-Mensah & De-Graft Aikins 2010; Maredza, Bertram & Tollman 2015). Aside from the physical consequences of stroke, stroke has a serious economic impact on the Ghanaian working population as most of the stroke patients in Ghana are within the working age (Agyemang et al. 2012).

The cardinal effect of stroke on its survivors is disability (George & Steinberg 2015). The long-term physical consequences of a stroke put a lot of burden on the stroke patients, their families, the healthcare system and the economy at large (Brewer et al. 2013). Stroke patients in Ghana are also burdened with debilitating impairments and functional deficits as expected of all stroke patients (Baatiema et al. 2017a; William et al. 2017). Effective rehabilitation is key to reducing and improving the level of disability as well as improving the quality of life of stroke patients (Hatem et al. 2016). Stroke patients in LMICs such as Ghana are still burdened with disability despite undergoing rehabilitation whereas stroke survivors in high-income countries (HICs) often may experience better functional and participation outcomes such as return to work (Rhoda et al. 2015; William et al. 2017). This could be attributed to a couple of factors of which one could be the quality of stroke rehabilitation services provided. Stroke patients are probably doing better in function in HICs because of the better quality in their stroke rehabilitation services (Bernhardt et al. 2020) including all the necessary structure and process of rehabilitation. Quality of stroke rehabilitation seems to be better in these HICs because of the availability of data and evaluation of stroke rehabilitation services. Studies that have been conducted to evaluate stroke rehabilitation services in HICs have provided recommendations for improvements which when implemented, enhances the structure and process of stroke care, which then translated into better patient outcomes. However, in LMICs such as Ghana, data are lacking on the evaluation of the available stroke rehabilitation to help inform policy development on stroke rehabilitation that could improve the overall quality of stroke care. Therefore, there is the need to fill this gap in the research evidence on stroke rehabilitation in Ghana. In order to do so, there is the need to evaluate the structure and process of stroke rehabilitation available in LMICs such as Ghana.

The quality of care directly influences the outcome of the stroke patients. Ameh and colleagues affirm that a good structure can promote good process and in turn, a good process can promote a good outcome (Ameh et al. 2017). The structure and process of healthcare are therefore very important when considering the quality of care which translates into the better patient outcomes. Structure is considered as the physical and organisational aspects of healthcare. These are factors that affect the context in which healthcare is provided (Hoenig et al. 2002). The structural component of healthcare takes into consideration the personnel by looking at their education, training, experience and certification. It also considers the setting where healthcare is provided, which includes systemic organisation, staffing and equipment availability, among others (Haj, Lamrini & Rais 2013). Processes are the activities implemented in the rehabilitation services of patients with disabling conditions that help to progress patients’ health by promoting recovery, functional restoration, survival and even patient satisfaction (Hoenig et al. 2002). These process-related factors may include diagnosis, interventions, education, preventive treatment, guidelines as well as procedures, coordination of care, individualisation, amount and timing and specific interventions (Donabedian 2003).

This study aims to provide data to help in better understanding of the structure and process of stroke rehabilitation in Ghana to help in facilitating interventions to improve the quality of stroke rehabilitation, which in turn can improve the outcome of stroke patients. A preliminary study carried out in Ghana to assess the structure and process of stroke rehabilitation in Ghana was conducted by the authors of this study (Mohammed, Nyante & Mothabeng 2022) using quantitative methods. In order to have a better understanding of the structure and process of stroke rehabilitation that was recorded in the previous quantitative study, a qualitative study needed to be carried out. This qualitative study hopes to further validate the results obtained from the quantitative study and also provide more details of the structure and process of stroke rehabilitation available. The qualitative study will provide in-depth information, which will help to enrich the literature on the structure and process of stroke rehabilitation in Ghana. This study therefore aimed to explore stroke healthcare professionals views and experiences of the structure and process of stroke rehabilitation in three selected hospitals in Ghana in order to ensure interventions are implemented to improve the quality of stroke rehabilitation services locally.

## Research methods and design

### Study design

This study employed a descriptive phenomenological approach to clearly assess the views and experiences of HCPs directly involved in the rehabilitation of stroke patients in Ghana. Phenomenology involves describing the experiences and views of a group of individuals about a particular concept or phenomenon, resulting in several people sharing their experiences on the same matter (Creswell 2009).

### Study population and sampling strategy

This study purposively sampled 26 stroke HCPs who were directly involved in the day-to-day rehabilitation of stroke patients from three selected hospitals in the Greater Accra Region of Ghana. Purposive sampling is a non-probabilistic sampling procedure used in sampling participants in qualitative studies. This sampling technique allows researchers to choose the sample based on who they think best fits to be part of the study (Crossman 2020). In this study, the researcher sampled a maximum of two stroke HCPs from each of the available rehabilitation professions at each of the three selected hospitals. The HCPs who were recruited included doctors, nurses, physiotherapists, occupational therapists, speech and language therapists, clinical psychologists and dieticians. These HCPs were selected if they met the following criteria:

- If they were 18 years and above
- If they had worked in stroke rehabilitation for at least 1 year or if they had any formal training or specialisation in stroke rehabilitation.

### Settings

This study was carried out at the general medical wards and stroke unit of three selected hospitals in the Greater Accra Region of Ghana. Greater Accra is the capital city of Ghana and most of the healthcare delivery settings are involved in rehabilitation. The selected sites represented all the levels of local healthcare and included a tertiary level hospital (TH) (Korle Bu Teaching Hospital), a secondary level hospital (SH) (Tema General Hospital) and a primary level hospital (PH) (Amasaman District Hospital). All the three levels of healthcare are involved in both inpatient and outpatient stroke rehabilitation.

### Data collection

The first author visited the medical wards and stroke unit of the hospitals to identify HCPs who met the study inclusion criteria. Participants were provided with copies of the study information sheet, which explained the aims as well as the procedures of the study. Participants had the opportunity to ask questions about the study and answers were provided. Written informed consent was provided by each participant who agreed to participate through signing of the consent form. Individual in-depth interviews were conducted in English at a mutually agreed venue and time at the medical and stroke units of the selected hospitals. These individual interviews were conducted in-person in a quiet room. The first author was the moderator of the interview. A semi-structured interview guide (Appendix 1) was used to guide the interviews. Before the start of each interview, the interviewer (first author) engaged participants in informal conversation to establish rapport and prepare them for the interview. The interview questions were focused on two main themes, which were the structure and process of the local stroke rehabilitation. The interview questions were developed based on review of literature and also with guidance from a previous quantitative study conducted by the authors. With the permission of the participants, each interview was audio recorded using a digital voice recorder. The interview lasted between 30 min and 1 h. During the interview, the researcher intermittently summarises the participants’ contributions to ensure that their views were accurately understood. The recorded interviews were transcribed verbatim and entered into a Microsoft Word document.

### Data analysis

Data analysis occurred concurrently with data collection as transcription was performed alongside data collection. Transcripts were assigned specific identification numbers. Analysis also included reading and re-reading of the transcripts to help generate codes by authors. A code book was then developed. Themes and sub-themes were generated to capture the codes. The first two authors worked together to develop codes. Themes and subthemes were then generated upon discussion among the authors. The third author cross-checked the coding and themes developed. The stages of thematic analysis served as a guide in the analysis process. NVivo software (QSR International company, Burlington, Massachusetts, United States) was used to manage the data.

### Ethical considerations

Ethical approval was received from the Ethical and Protocol Review Committee of School of Healthcare Sciences, University of Pretoria (protocol no.: 68/2020), Ghana Health Service Ethics Review Committee (protocol no.: GHS-ERC 010/02/20) and Korle Bu Teaching Hospital Ethical and Protocol Review Committee (protocol no.: KBTH-IRB/000165/2019). Permission was sought from the heads of the hospitals and the departments where data were collected. Written informed consent was sought from each of the participant. Participants’ confidentiality and anonymity were assured. All coronavirus disease 2019 (COVID-19) safety measures were duly observed.

## Results

The interviews conducted in this study included questions on the available structure and process of stroke rehabilitation, which gave rise to themes and sub-themes. Two main themes that were structure and process of stroke rehabilitation emerged with several sub-themes under each theme. The sub-themes for the structure of stroke rehabilitation included rehabilitation unit, bed capacity, approach of care, rehabilitation protocol, staff capacity development and payment system. For the process of stroke rehabilitation, the sub-themes that emerged were frequency of rehabilitation, duration of rehabilitation, length of hospital stay and discharge process, post-discharge care and follow-up and family or relative involvement in rehabilitation.

### Participants

Twenty-six HCPs were recruited from the three selected hospitals. Of the total participants, there were six physiotherapists, two occupational therapists, two speech therapists, six nurses, two dieticians, two clinical psychologists and six medical doctors as shown in Table 1. Majority of participants (65.4%) were females with 34.6% being males. Table 1 shows the lack of some HCPs at the PH and SH, which were occupational therapists, speech therapist and clinical psychologists.

**TABLE 1:** Profession of participants.

Profession	Tertiary level hospital	Secondary level hospital	Primary level hospital
Nurse	2	2	2
Physiotherapist	2	2	2
Occupational therapist	2	0	0
Speech therapist	2	0	0
Clinical psychologist	2	0	0
Medical doctor	2	2	2
Dietician	1	1	0

**Total**	**13**	**7**	**6**

### Structure of stroke rehabilitation

#### Stroke rehabilitation unit

The participants revealed that rehabilitation of stroke patients was carried out in general medical wards and stroke unit in the TH. However, at the SH and the PH, respondents mentioned that stroke patients were rehabilitated at the general medical wards only. At the TH, some respondents mentioned that admission of stroke patients into either the medical wards or the stroke unit was mostly dependent on the availability of beds and the severity of the stroke:

‘Yes, normally what we do is, when the patient is brought to the emergency and there is space at the stroke unit, they are admitted there straight. But mostly the acute ones. And if there is a first-time stroke patient too, we admit at the stroke unit. But if there is no bed available, we admit at the main ward.’ (Participant 6, TH, Nurse)

Participants across all the rehabilitation settings recommended a stroke unit as the most effective setting for rehabilitation of stroke patients:

‘Of course, I will recommend the stroke unit.’ (Participant 7, TH, Psychologist)

‘Obviously if there was a separate section for stroke patients, then certainly the management will be enhanced.’ (Participant 18, PH, Nurse)

‘In a nutshell, what I’m recommending is that we should all have stroke units, you see? But with the requisite staffing, education, availability of extra beds.’ (Participant 25, SH, Doctor)

#### Bed capacity for stroke rehabilitation

Participants across all the three hospitals mentioned that there was inadequate bed capacity for rehabilitation of stroke patients. The inadequacy in bed capacity at the stroke unit of the TH was reported as the reason some of the stroke patients were admitted to the general medical wards. participants mentioned that the PH and SH had no designated beds for stroke rehabilitation:

‘Not at all, we do not have adequate bed capacities. Sorry to cut you short, but I mean it’s a straight no! When you discharge somebody in the morning, the next 30 minutes to 1 hour, another person is on that bed.’ (Participant 26, PH, Physiotherapist)

‘No. We don’t even have the capacity. We don’t really have beds for stroke patients.’ (Participant 14, SH, Nurse)

‘Well, we (at the stroke unit) have a few beds, so sometimes we are forced to admit some patients at the medical ward or any ward provided the bed there is suitable to accommodate the patient.’ (Participant 6, TH, Nurse)

#### Approach of care for stroke rehabilitation

Participants from the TH mentioned the use of a multidisciplinary team (MDT) approach to stroke rehabilitation. However, this MDT approach to care was only available at the stroke unit. In the PH and SH, the MDT approach to care was reported not to be available as stroke patients were managed with other medical cases:

‘No. We don’t come together as a team. Ideally, that would have been good, so that in the morning when we are doing our rounds, we have the pharmacist around, the physiotherapist around, the dietician around. That would be very ideal. But unfortunately, we cannot do that here. Here, everything has different schedules. Our numbers are not enough to be able to put key members together to form the stroke team. What we however do is, the physicians do their rounds first. When they finish their rounds, they check the blood pressure and make sure the patients are fine and there’s no issue. Then we write a referral note for the dietician to come and see and play his part before the physiotherapist will come and see and also play his part. So, at the end of the day, everybody gets to see the patient. So, we don’t all go together as a team but we benefit from each team’s expertise.’ (Participant 25, SH, Doctor)

‘There’s nothing like a stroke rehab team; we work separately.’ (Participant 19, PH, Physiotherapist)

‘You know, you need a team to work. So, if you are alone, you can do a few things but you might not be able to do it properly. So, they might not be able to nurse them properly at the medical ward as compared to what we are doing at the stroke unit.’ (Participant 5, TH, Nurse)

#### Stroke rehabilitation protocol

Participants from some professions at the TH mentioned having site-specific protocols for stroke rehabilitation although not visibly displayed in the hospital. At the PH, protocols for stroke rehabilitation were displayed at the emergency unit and the general medical wards, which served as guide for the nursing staff specifically. The SH was reported to have site-specific protocol for general management of medical cases, but not for stroke management:

‘[…] Usually the in-charge of the unit has the protocol but usually they will educate you. You are oriented. But I have not seen the document. But it is there.’ (Participant 2, TH, Physiotherapist)

‘Yes, we have protocols that we have modified.’ (Participant 11, TH, Dietitian)

‘It’s just a general medical protocol. So, I think if we have a protocol for stroke management, I think it will be very helpful.’ (Participant 14, SH, Nurse)

#### Staff capacity development

Participants from the TH mentioned that there was the availability of staff capacity development programmes at the stroke unit. These programmes involved weekly meetings to learn more on stroke rehabilitation through discussions and presentations. Staff capacity development programmes for stroke rehabilitation were reported not to be available at the general medical wards of the TH, SH and PH:

‘Normally after our MDTs we do our presentations. So maybe this week it will be the turn of the nurses, the doctors and then we pick other few topics and then discuss, and then do a whole presentation.’ (Participant 5, TH, Nurse)

The majority of the participants acknowledged the need for continuous education programmes related to stroke rehabilitation for staff, which were not available at their facilities:

‘Most staffs should be trained on how best we can handle our stroke patients. It would be very helpful.’ (Participant 21, PH, Nurse)

‘The first thing will be regular training, regular training.’ (Participant 24, SH, Dietitian)

#### Payment for stroke rehabilitation

All participants from the TH mentioned that stroke patients paid for rehabilitation services out of pocket at the stroke unit. For stroke patients managed in the general medical wards, the payment system allowed for the use of the national health insurance scheme (NHIS) for some rehabilitation services at all the hospitals. Also, it was revealed by some participants that ability to pay for rehabilitation services was also a factor for admission of stroke patients either to the stroke unit or general medical ward at the TH:

‘Most patients here (in the stroke unit) who are discharged its cash and carry. But the Medical Ward is covered by Health Insurance and this is even a factor for placing patients.’ (Participant 5, TH, Nurse)

The NHIS did not cover stroke rehabilitation services such as physiotherapy for inpatient rehabilitation. Participants recommended that stroke rehabilitation should be completely covered by the NHIS:

‘Ok. So, I can speak for physio. If they can advocate for physio inpatient. Presently, outpatient is catered for by the national health insurance. But inpatient, insurance does not cater for.’ (Participant 17, SH, Physiotherapist)

‘It’s more and less like cash and carry. You’ll pay, then when you pay, I attend to you. it’s not helpful because, right from the onset when you tell them they’re supposed to pay for the service, they don’t appreciate that idea. This is because, they feel the medical service is being paid for by the N.H.I.S.’ (Participant 26, PH, Physiotherapist)

### Process of stroke rehabilitation

#### Frequency of rehabilitation sessions

The number of rehabilitation sessions patients received was found to be similar in all the hospitals where stroke patients received rehabilitation once a day for five days. However, at the stroke unit, it was revealed from the responses that rehabilitation sessions could occur more than once per day depending on the patient’s needs. This was possible because the various HCPs were resident at the stroke unit. It was reported by the HCPs that the number of rehabilitation sessions received per week by patients at the general medical wards was based on affordability:

‘Okay. So, for stroke unit per week, we come to work from Monday to Friday. So, from Monday to Friday, we treat for the stroke unit. But for the medical unit, first of all, we give you a bill for treatment session. Or sometimes even if we see that you can pay, we just give you the bill for the week. So, we can give you five sessions of therapy within the week. But normally we start with three sessions. Because of affordability we cannot know whether the patient can. So, you pay for three sessions. Within the week, we do the three sessions for you. After that if you need more, we write another session for you to go and pay.’ (Participant 2, TH, Physiotherapist)

#### Duration for sessions

A minimum of 30 min – 45 min of rehabilitation was reported for patients at all the three hospitals but stroke patients in the stroke units were reported to receive more rehabilitation time because of staff availability.

#### Availability of computed tomography scan and magnetic resonance imaging

Computed tomography (CT) scan and magnetic resonance imaging (MRI) availability in the facilities were reported by participants to be of high relevance to the rehabilitation of stroke patients as they help in diagnosis and also help guide stroke management. Even though this was found to be of much relevance, only TH was equipped with these:

‘Yes. Basically, it helps us to determine which type of stroke it is and the extent to which the stroke has occurred. I think they should be done as early as possible after you’ve done your physical examination and make the diagnosis. That’s the next thing you should be. Unfortunately, we don’t have in-house CT scan machine or MRI machines.’ (Participant 25, SH, Doctor)

‘The unfortunate thing is that we don’t have. So, for most of our situation, we usually have to manage for the first two or three days without a definite diagnosis which is quite daunting because you have to really hedge. But when it happens that we have to take a scan, we have facilities around where we can do the scan.’ (Participant 22, PH, Doctor)

#### Length of hospital stay and process of discharge

Discharge of stroke patients was reported by all participants to be carried out by doctors. For all the hospitals, participants mentioned that stroke patients were discharged from inpatient rehabilitation on the basis of medical stability to continue their rehabilitation on an outpatient basis. Stroke patients at the TH were reported to be discharged within 2–14 days of admission. At the SH and PH, patients were discharged within 3–4 days of admission:

‘And so, we [*doctors*] discharge the patient when we are sure that the home care of the patient can be managed by those either at home or at a nursing care and that the patient no longer has urgent needs in which we need to manage at the ward. We have an average from between two to fourteen days but we give that by the tenth they should have gone home.’ (Participant 9, TH, Doctor)

#### Post-discharge care and follow-up

Participants from all the three hospitals reported that stroke patients who were discharged usually go home to continue rehabilitation on an outpatient basis. There were no follow-up visits by HCPs to patient homes although some of the HCPs mentioned that follow-up visits to patients’ homes could help monitor the patients’ condition and progress of improvement. It was also reported that some stroke patients requested for home follow-up visits to their homes and these follow-up visits were however reported to be at the patient’s affordability:

‘It would be better if we do follow ups, because most cases you discharge them stable. But when they come back, most of them might have changed and gone worst. So, if they can do the follow ups, it would be better.’ (Participant 20, PH, Doctor)

‘It would be better if we could follow-up at home. That will be best, that will be best. If it’s possible. Because you know, the logistical aspect comes in.’ (Participant 24, SH, Dietitian)

‘So, they continue to have regular reviews at the stroke unit. Some of them opt to have homecare. But that one, of course, you have to pay for a doctor to come home; unless of course, they have a nurse at home and the nurse will be monitoring them so that, if any issues come up, the nurse would communicate to the doctor. But this has to be a personal idea, it is not sponsored by the hospital.’ (Participant 1, TH, Physiotherapist)

#### Family or relatives involvement in stroke rehabilitation

Participants from all the hospitals mentioned the involvement of family or relatives in the rehabilitation process of the stroke patients. The participants acknowledged the relevance and the benefits of involving patients’ and their families in the rehabilitation as this helps the patients and their relatives to understand the condition as well as the treatment being given:

‘It has been very beneficial because we involve the relatives from the first day. We talk to them, give them an insight into the condition so they don’t panic. They get to understand what is happening and how to relate with their patients who is been brought here and also if there is anything, because we engage them.’ (Participant 4, TH, Occupational therapist)

‘For stroke patients, their relatives are always available. We don’t let all of them go and leave the patients.’ (Participant 19, PH, Physiotherapist)

‘Very, very. The caregivers do a lot. Some come here and they would want to bring all the meals from home. For those people, we start engaging the caregivers right from the beginning. Right from the beginning, what they can bring, what they cannot bring. There, we work with them, go and observe the food they bring from the kitchen, from the house and all those ones. So, they are crucial. In fact, I don’t know how we would be able to make any impact in the stroke cases without caregivers.’ (Participant 24, SH, Dietitian)

## Discussion

The structure and process of stroke rehabilitation form a very important aspect of rehabilitation that can affect the outcome of stroke survivors. This aspect of rehabilitation however remains understudied especially in resource-limited settings such as Ghana. This study aimed at exploring stroke HCPs views and experiences of the structure and process of stroke rehabilitation in thes Greater Accra Region of Ghana. This study found some limitations in the structure and process of stroke rehabilitation in Ghana although there were some services that were available as reported by participants.

Majority of stroke patients were reported in this study to be rehabilitated in general medical wards. Ideally, stroke patients are expected to be rehabilitated in designated stroke units as recommended internationally (Chimatiro & Rhoda 2019). Outcome of stroke patients managed in designated stroke units has been reported to be better than those managed in general medical wards (Adams et al. 2003; Langhorne et al. 2002). The stroke unit system of rehabilitation has been adopted internationally, especially by HICs with good success rates (Christian et al. 2016; Gould et al. 2011; Ras 2009). However, LMICs such as Ghana are yet to adopt this stroke unit system of rehabilitation as there is only one stroke unit in Ghana as recorded in this study and previous studies (Baatiema et al. 2017a; Sanuade et al. 2021). The first and only stroke unit in Ghana was established in January 2014 in collaboration with a health team from Wessex in the United Kingdom (UK). To date, this stroke unit continues to remain the only stroke unit in Ghana (Baatiema et al. 2017a). A recent review by Wasti et al. (2021) also confirmed the lack of a well-structured stroke rehabilitation system in LMICs such as Ghana. The lack of roll-out of more stroke units since the establishment of the first one shows that stroke is not well prioritised in Ghana despite its increasing prevalence. The HCPs in this study also recommended the rehabilitation of stroke patients in a well-equipped dedicated stroke unit to help enhance the management of the stroke patients, which will in turn improve their functional outcome.

This study also recorded limited bed capacity for stroke rehabilitation across the hospitals as reported by participants. At the PH and SH, this study found that there were no designated beds for stroke patients. Stroke patients would therefore have to compete with other medical cases for beds in the general medical wards. Although there was a stroke unit at the TH, participants also reported limited bed capacity, as reported in similar studies in Ghana (Baatiema et al. 2017a; Morris 2011). As stated by some of the TH participants, limited bed capacity was one of the reasons why some stroke patients were also rehabilitated in the general medical wards, indicating that only a small percentage of Ghanaian stroke patients were able to access the stroke unit. Sanuade et al. (2021) reported that the limitation in bed capacity for stroke rehabilitation in Ghana sometimes delayed the start of the rehabilitation and this tend to affect the outcome of rehabilitation negatively.

The MDT approach to care for stroke patients has been recommended to be the best approach to stroke care, which improves the outcome of stroke patients (Clarke 2013). During the Wessex Ghana Stroke Partnership, the MDT approach of care for stroke was recommended by the UK team as a very important tool for effective stroke care (Johnson et al. 2017). This MDT approach to care has been found in this study to be practiced at the stroke unit of the TH only. However, the PH and SH as well as the medical wards of the TH did not practice the MDT approach to care for stroke despite its known benefit. Baatiema et al. (2017b) also reported the lack of MDT approach to care for stroke management in various hospitals across Ghana. The possible reasons why the PH and SH did not practice the MDT approach to care could be because of the lack of a dedicated stroke unit and the unavailability of some HCPs. It was also found in this study that the PH and SH did not have the services of occupational therapists, speech therapists and clinical psychologists. Therefore, stroke patients in these facilities did not have access to the services of these HCPs showing a limitation in their rehabilitation, which could affect their outcome as also reported by Ameh et al. (2017). Similar studies conducted in Ghana revealed the unavailability of some HCPs for stroke rehabilitation especially the allied health professionals, which included occupational therapists, speech therapists and clinical psychologists (Baatiema et al. 2017b; Sanuade et al. 2021; names deleted to maintain the integrity of the review process).

Capacity development for staff in stroke rehabilitation is key to rehabilitation as it helps the HCPs be more abreast with current management strategies and methods as well as evidenced-based practice. Staff capacity development is crucial to stroke care as it contributes to the quality of care through equipping the HCPs with current evidence-based knowledge and skills in stroke care (Baatiema et al. 2017a). Staff capacity development was found in this study to only be available at the stroke unit of the TH and this was because the unit only managed stroke patients. However, for hospitals without a designated stroke unit, developing staff capacity in stroke care only might be difficult as the same HCPs managed stroke alongside other medical cases. A similar study conducted in Ghana on the barriers of stroke care confirmed the lack of staff capacity development in stroke management (Baatiema et al. 2017b).

This study found that stroke rehabilitation services were mostly paid out of pocket and not fully covered by the NHIS as also recorded in the review by Ekeh (2017). The NHIS was introduced in Ghana in 2003 to help reduce the financial burden of diseases and health on Ghanaians (Gould et al. 2011). However, the coverage of the national health insurance is limited especially for stroke rehabilitation. Therefore, stroke patients who were not financially stable might not have access to rehabilitation services, which are not covered by the NHIS. Anecdotal information reveals that there are ongoing dialogues to help get all stroke rehabilitation services on the health insurance scheme and this will be of utmost benefit to stroke patients as most of them are unable to afford rehabilitation services out of pocket.

The frequency and duration of rehabilitation was found to be similar in all the hospitals in this study. However, for stroke rehabilitation services such as physiotherapy, the frequency differed among patients across the three hospitals depending on the affordability of the therapies as they were not covered by the national health insurance. Based on the affordability some patients received less therapy sessions per week.

Computed tomography and magnetic resonance imaging scanning were reported by respondents in this study to be of high relevance to the rehabilitation of stroke patients as they help in clinical decision making on the approach and type of rehabilitation to be carried out. These special investigations were not available at the primary and secondary hospitals in this study. Another study in Ghana also reported the limitation with equipment for diagnosis of stroke, which include CT and MRI scanning (Sanuade et al. 2021). The unavailability of these equipment tends to delay the rehabilitation process as patients were often referred to other facilities for the scans to be carried out as reported by some respondents. According to Murie-Fernández et al. (2012), as rehabilitation delays, the complications of the stroke worsen, affecting the outcome of the stroke patient.

It has been recommended that discharge of stroke survivors from inpatient rehabilitation should be planned by the MDT (Wasti et al. 2021). However, in this study, discharge from acute in-patient care was planned and carried out by the medical doctors and the basis for discharge was medical stability. Recommendations based on existing guidelines for discharge of stroke patients from acute care are when patients have gained medical stability and have also gained some form of functional independence (Winstein et al. 2016). Stroke patients in Ghana were reported in this study to be discharged from inpatient rehabilitation within 14 days of admission showing a shorter length of hospital stay. A study conducted in Ghana also reported that stroke patients in Ghana have shorter length of hospital stay (Mohammed, Nyante & Mothabeng 2022). One possible reason for the early discharge could be because of the limitation in bed capacity for stroke rehabilitation. Stroke patients were discharged to continue rehabilitation on an outpatient basis to make room for new admissions.

This study also found that the only outpatient services available for stroke patients in Ghana were the regular medical check-ups and physiotherapy. Ideally, outpatient stroke rehabilitation should be designed to provide multidisciplinary rehabilitation, which includes all the core stroke rehabilitation disciplines such as the doctors, nurses and therapists as recommended in previous studies (Janzen et al. 2019; Wasti et al. 2021). This study further found that there were no follow-up visits to patients’ home and communities after discharge from hospital inpatient rehabilitation, which was also reported in a previous study by Tinney et al. (2007). Discharge of stroke patients from acute inpatient care to home and community-based rehabilitation, in addition to the outpatient rehabilitation helps in achieving good outcome for stroke patients because of the continuity of care to prevent secondary complications and to promote community reintegration. Community-based rehabilitation is effective in tackling issues such as return to work and activities of daily living, which involve patients and their families in the rehabilitation process (Walker, Sunnerhagen & Fisher 2013). Community-based rehabilitation includes the social and family support in rehabilitation, which in turn, provides the necessary physical, emotional and spiritual support needed by the patient (Wasti et al. 2021). This study as well as that of Sanuade et al. (2021) recommend the introduction of home and community-based stroke rehabilitation in Ghana to help minimise the complications of stroke as well as to reintegrate patients back into their previous life.

### Limitations and recommendations

This study used a qualitative method that makes generalisability of the obtained data limited. The data obtained from this study are limited to the settings where data were collected. This study therefore recommends conducting similar studies in other settings across the country in order to have more data across the country on the structure and process of stroke rehabilitation.

## Conclusion

This study explored the perception of HCPs on the available structure and process of stoke rehabilitation in the Greater Accra Region of Ghana and through this exploration, some limitations of the service were recorded. There were also some of the elements of structure and process that were found to be available for stroke rehabilitation. For the structure of rehabilitation, stroke unit and the use of an MDT approach to care were only available at the tertiary hospital. All hospitals recorded limited bed capacity, a lack of rehabilitation protocols and payment of rehabilitation services out of pocket. This study also recorded a lack of staff capacity development for staff who manage stroke patients in general medical wards. For the process of rehabilitation, most stroke patients received rehabilitation, mainly physiotherapy, five times a week for about 30 min – 45 min. CT and MRI scanning were only available at the tertiary hospital. Patients were also discharged by doctors when they are medically stable and referred to continue rehabilitation on an outpatient basis. These reported gaps and limitations in stroke-related services highlighted the need for improvement in the structure and process of stroke rehabilitation in order to ensure quality of stroke care for the stroke patients.

## Appendix 1

### Interview guide

#### Structure questions

1. Think about the structure of acute stroke rehabilitation in this hospital.
2. What are the units of rehabilitation in this hospital?
  **Probe:**
  - General medical wards and stroke unit
  - What criteria is used to inform which patient is admitted in general medical ward or stroke unit?
  - Based on your experience, how different is the stroke unit management from the general medical ward management.
  - Based on your experience, which is more effective and which will you recommend and why?
3. What is the bed capacity for the units of rehabilitation?
  **Probe:**
  - Are the number of beds for stroke rehabilitation enough? (for both stroke unit and general medical wards). Any recommendations?
4. What approach of care is used in stroke rehabilitation?
  **Probe:**
  - Is there an MDT approach of care?
  - If there is, what are the constituents of the MDT?
  - Which of the HCPs do you think are not part of the team and how relevant are they to the rehabilitation of the stroke patients?
  - Are the HCPs actually working together as a team or they work in segregation?
  - If they work in segregation, how is this affecting patient management? What will you recommend based on you experience?
5. Are HCPs specifically trained in stroke rehabilitation or they are general practitioners?
6. How frequently do staff continuous education take place?
  **Probe:**
  - How helpful is this programme?
  - Any recommendation?
7. What guides the rehabilitation of stroke?
  **Probe:**
  - Is there a written down protocol or guideline for stroke rehabilitation?
  - If there is, is it actually used and how helpful is it?
  - Are resources available to execute what is in the guideline or protocol?
  - Do you think it conforms with recommended standards?
  - Do you think there should be adjustments to the protocol? Any recommendations?
8. How do patients for rehabilitation services?
  **Probe:**
  - Is this payment system the best?
  - What do you recommend for stroke rehabilitation and why?
  - Any recommendations?

**Process questions**

1. Think about the available process of acute stroke rehabilitation in this hospital
2. How many therapy sessions do patients receive per week?
  **Probe:**
  - Are the therapy sessions received by stroke patients enough?
  - Do you think more sessions could be done and why?
  - Any suggestions?
3. What is the duration of rehabilitation for each session?
  **Probe:**
  - Is this enough?
  - Any recommendations?
4. How relevant is CT scan and MRI availability onsite?
  **Probe:**
  - How helpful are these to rehabilitation of the stroke patient?
5. What is the length of hospital stay for stroke patients?
  **Probe:**
  - Based on your experience of stroke rehabilitation, do you think it is enough for patient recovery?
  - If it is short, what could be the possible reasons for this?
  - Any recommendations?
6. Based on your experience of stroke care what do you think the basis for discharge from acute care should be?
  **Probe:**
  - Do you think they receive adequate care before they are discharged?
  - Any reasons for the basis of discharge?
  - Any suggestions?
7. What are patients discharge destination?
  **Probe:**
  - Is follow-up to home and community part of the patient discharge plan?
  - Any recommendations?
8. What are the outpatient destinations for rehabilitation? Any recommendations?
9. At what point is the patient and family part of the decision making for patient management?

### General recommendations

1. What recommendations will you like to make to the hospital authorities on how to improve services for stroke rehabilitation in terms of the available structure and process in order to help improve patient outcome after stroke.
2. Are there any other contributions you will like to share on this issue discussed before we come to the end of this discussion?
